# The Chemical Compositions, and Antibacterial and Antioxidant Activities of Four Types of Citrus Essential Oils

**DOI:** 10.3390/molecules26113412

**Published:** 2021-06-04

**Authors:** Xiaocai Lin, Shan Cao, Jingyu Sun, Dongliang Lu, Balian Zhong, Jiong Chun

**Affiliations:** 1 National Navel Orange Engineering Research Center, College of Life Sciences, Gannan Normal University, Ganzhou 341000, China; GNNUlxc18879862383@163.com (X.L.); scoral29116@163.com (S.C.); SJYnj_1997@163.com (J.S.); bal.zh@163.com (B.Z.); 2 College of Chemistry, Gannan Normal University, Ganzhou 341000, China; ludl201306@163.com

**Keywords:** citrus, essential oil, GC-MS, antibacterial, antioxidant

## Abstract

Nanfeng mandarins (*Citrus reticulata* Blanco cv. Kinokuni), Xunwu mandarins (*Citrus reticulata* Blanco), Yangshuo kumquats (*Citrus japonica* Thunb) and physiologically dropped navel oranges (*Citrus sinensis* Osbeck cv. Newhall) were used as materials to extract peel essential oils (EOs) via hydrodistillation. The chemical composition, and antibacterial and antioxidant activities of the EOs were investigated. GC-MS analysis showed that monoterpene hydrocarbons were the major components and limonene was the predominate compound for all citrus EOs. The antibacterial testing of EOs against five different bacteria (*Bacillus subtilis*, *Staphylococcus aureus*, *Escherichia coli*, *Pseudomonas aeruginosa* and *Salmonella typhimurium*) was carried out using the filter paper method and the broth microdilution method. Kumquat EO had the best inhibitory effect on *B. subtilis*, *E. coli* and *S. typhimurium* with MIC (minimum inhibitory concentration) values of 1.56, 1.56 and 6.25 µL/mL, respectively. All citrus EOs showed the antioxidant activity of scavenging DPPH and ABTS free radicals in a dose-dependent manner. Nanfeng mandarin EO presented the best antioxidant activity, with *IC*_50_ values of 15.20 mg/mL for the DPPH assay and 0.80 mg/mL for the ABTS assay. The results also showed that the antibacterial activities of EOs might not be related to their antioxidant activities.

## 1. Introduction

Citrus trees are among the most abundant crops in the world and are widely grown in tropical and subtropical regions [1]. Citrus trees belong to the Rutaceae family, which has approximately 140 genera and 1300 species [2]. China is one of the world’s largest producers of citrus fruit, having produced 23.1 million tons of tangerines/mandarins (70% of the global production) and 7.5 million tons of oranges (15% of the global production) in 2020 [3]. Jiangxi Province in southern China is one of the largest citrus production regions. The navel orange (*Citrus sinensis* Osbeck cv. Newhall), Nanfeng mandarin (*Citrus reticulata* Blanco cv. Kinokuni), Xunwu mandarin (*Citrus reticulata* Blanco) and Yangshuo kumquat (*Citrus japonica* Thunb) are the main local citrus varieties of Jiangxi Province and are deeply loved by consumers. The Nanfeng mandarin was originally produced in Nanfeng County, which is famous for its fruit and has a long history of cultivation. It has thin skin, juicy flesh and a rich aroma, making it an important international trade commodity [4]. The Xunwu mandarin is a special citrus variety of Xunwu County of Ganzhou City in Jiangxi Province, which is also a famous cultivation area. The kumquat is produced by small fruit trees in the Rutaceae family, which have been widely cultivated in many countries in recent years [5,6]. It is excellent source of nutrients and phytochemicals, including ascorbic acid, carotenoids, flavonoids and essential oils (EOs), which are often used in traditional medicine [7,8]. Ganzhou City in Jiangxi Province is the main region for the production of navel oranges in China, and the Newhall navel orange is the predominant variety, accounting for over 80% of the total product [9,10]. Newhall navel orange trees produce large amounts of fruit that are subject to physiological fruit drop each year [11]. Said fruit is generally discarded. It is important to extract valuable products from the dropped fruit to avoid wastage of natural resources [12].

The citrus processing industry and fresh consumption generate large amounts of citrus peel waste every year, accounting for nearly 50% of wet fruits. It is very important to develop methods for the conversion of these peel wastes into value-added products. Since citrus peels are rich in EOs, extracting EOs and exploring applications for them is one of the best ways to utilize this natural resource [13]. At present, citrus EOs have become the focus of many studies for their chemical components and biological activities—antibacterial, antioxidant, anticancer, insecticidal, etc. [14,15,16]. The antibacterial and antioxidant activities of citrus EOs have been extensively studied. Frassinett et al. have shown that EOs of the bitter orange, the sweet orange, lemons and broad-skin citrus fruit have good antibacterial and antioxidant activities [17]. Djenane et al. studied the antibacterial activity and antioxidant activity of oranges, lemons and bergamot peel EOs [18]. Yi et al. extracted Nanfeng mandarin EO by mechanical pressing, identified 64 components and found that it has a broad-spectrum antibacterial effect and moderate antioxidant activity [19]. There have been many studies on kumquat EO. Wang et al. identified 25 components of kumquat EO, and showed that kumquat EO had obvious activity against bacteria and fungi [8]. Al-Saman et al. studied the chemical components, and antibacterial and antioxidant activities of kumquat EO [20]. The yields, chemical compositions and biological characteristics of EOs are affected by many factors, including variety, place of production and extraction method [21]. However, studies on the Nanfeng mandarin EO (NMEO), Xunwu mandarin EO (XMEO), Yangshuo kumquat EO (YKEO) and physiological drop of Gannan Newhall navel orange EO (PDEO) are still lacking. At present, the research on the biologically active substances of citrus physiological fruit drops mainly focuses on flavonoids, limonoids, phenolic acids, etc. [11,12]. As far as we know, there are no literature reports about PDEO and XMEO.

Citrus EOs have broad-spectrum antibacterial properties [22]. They can be used as natural substitutes for chemical fungicides and antibacterial drugs. They are also used in food preservation, and the prevention and treatment of animal and plant diseases caused by microbial pathogens [16]. The antioxidants of natural EOs can extend the shelf lives of foods [23]. Kraśniewska et al. found a two-component mixture of Spanish origanum oil and marjoram oil can protect minimally processed vegetables against *Listeria monocytogenes* to maintain peak sensorial quality [24]. EOs have the effect of scavenging free radicals and may play important roles in preventing brain dysfunction, cancer, heart disease and immune system decline [25,26]. Citrus EOs can be used as natural antioxidants in the food and pharmaceutical industries with broad application prospects [27,28]. Our study involved comparative studies on the chemical components of four citrus EOs in southern China. We also evaluated their antibacterial and antioxidant activities. We expect this research to provide a reference for the development and utilization of these citrus EOs.

## 2. Results and Discussion

### 2.1. Chemical Compositions of the Citrus EOs

**Figure 1:**
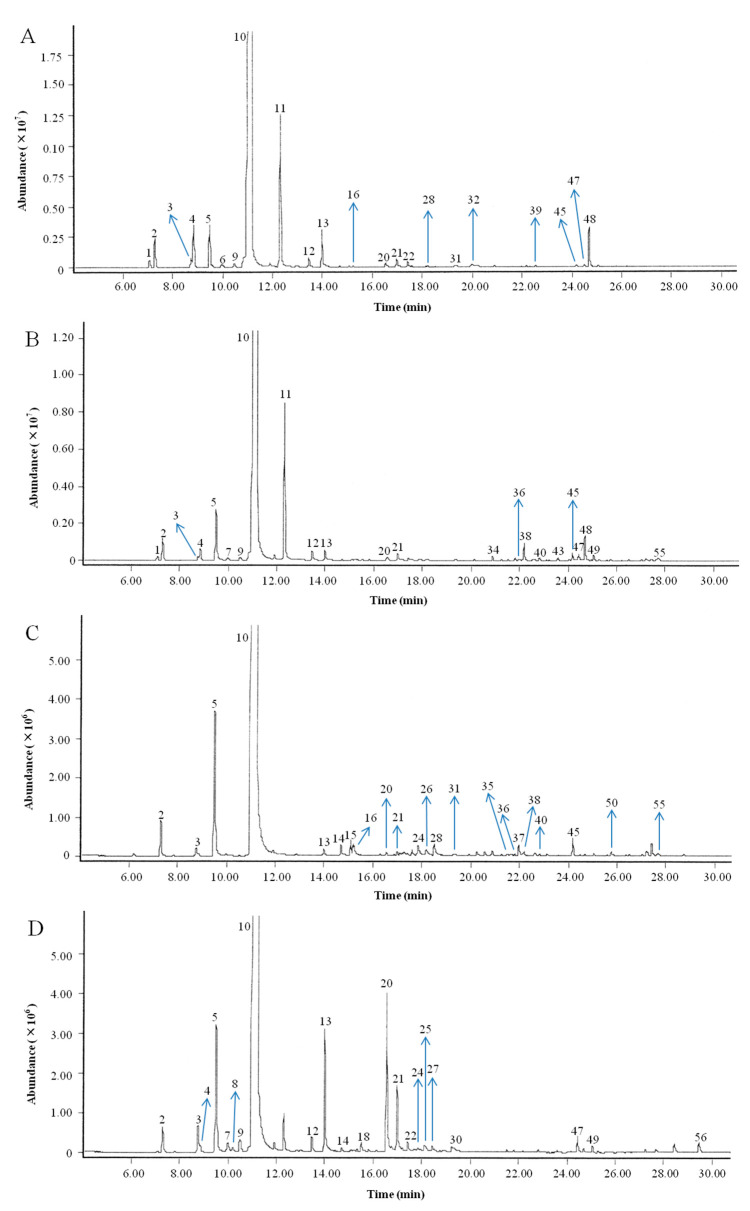
Total ion chromatograms of the four citrus EOs. (**A**) Nanfeng mandarin EO (NMEO). (**B**) Xunwu mandarin EO (XMEO). (**C**) Yangshuo kumquat EO (YKEO). (**D**) Physiological drop of Gannan Newhall navel orange EO (PDEO).

The chemical compositions of four citrus Eos were analyzed by GC-MS. The total ion chromatograms (TIC) of the EOs are shown in Figure 1. The relative content of each component was calculated from the total ion chromatogram according to the peak area normalization method. The components were identified according to a retention index, the NIST mass spectral library and the corresponding data published in the literature. Table 1 shows the analytical results for the four EOs. The number of individual compounds that comprise at least 0.05% of the total mixture (from Table 1) is marked above each peak in Figure 1.

As shown in Table 1, monoterpene hydrocarbons represented the most abundant components in all EOs: NMEO (93.96%), XMEO (95.94%), YKEO (95.04%) and PDEO (91.59%), respectively. PDEO had the highest (5.55%) and XMEO the lowest (0.95%) oxygenated monoterpene content. The contents of sesquiterpenes (0.41–2.33%) and oxygenated sesquiterpenes (0.03–0.23%) were relatively low. Limonene was the predominant compound in all citrus EOs. NMEO had a much higher content of straight-chain aliphatic aldehydes (0.95%) than the three other citrus EOs, including octanal (0.29%), decanal (0.57%) and dodecanal (0.09%).

Twenty-eight compounds were identified in NMEO, accounting for 99.63% of the total oil. Monoterpenes were the major components, accounting for 93.96% of the total oil. Limonene (79.13%) was the predominant component of NMEO, followed by γ-terpinene (8.19%), β-myrcene (2.08%), β-pinene (2.00%), linalool (1.81%), α-farnesene (1.50%) and α-pinene (1.22%). Yi et al. [19] studied the chemical composition of mechanical-pressed NMEO and found that the top three highest components were limonene (56.76%), β-pinene (12.10%) and γ-terpinene (12.03%), respectively. These differences may be at-tributed to different extraction method.

Thirty-three compounds were identified in XMEO, accounting for 99.41% of the total oil. Limonene (86.03%), γ-terpinene (5.80%) and β-myrcene (2.02%) were the main com-ponents. However, linalool, a very important component of citrus EOs, had a lower pro-portion in XMEO (0.43%) than in NMEO (1.81%). When checking the international standard for cold pressed, Italian type mandarin EO (Citrus reticulata Blanco) from ISO 3625 [29], we noticed this mandarin EO has a limonene content of 65–75%, γ-terpinene 16–22% and myrcene 1.4–2%. The differences between the Italian type EO and XMEO and NMEO may be caused by different production areas and extraction methods.

In YKEO, 28 components were identified, accounting for 98.10% of the total oil. Limonene (91.54%) and β-myrcene (2.73%) were the main compounds. Choi [30] detected the constituents of the cold-pressed peel oil of kumquats and found limonene (93.73%) and myrcene (1.84%) were the top two components of kumquat EO collected from Jeolla province, Korea. Wang et al. [8] found that the limonene content was only 74.79% in the hydrodistilled EO from kumquat (Fortunella crassifolia Swingle) peel; however, their myrcene content (7.11%) was much higher than in our YKEO. Linalool content (0.12%) was also very low in YKEO compared with NMEO (1.81%). The compositions of hydrodistilled samples of citrus EO vary according to genetic differences, soil and weather types, maturity stages, culturing conditions, etc. [31].

PDEO showed the presence of 37 components, accounting for 98.05% of the total EO. Limonene (88.25%), terpinen-4-ol (1.98%), β-myrcene (1.90%) and linalool (1.50%) were the principal components. In one report [32], cold pressed mature Gannan Newhall navel orange EO (NOEO) had similar limonene (85.32%) and linalool (1.29%) contents to PDEO, but far higher β-myrcene content (5.11%) than PDEO due to different maturity stages and extraction methods.

### 2.2. Antibacterial Activity

Citrus EOs have shown a wide spectrum of antimicrobial activities in vitro [22]. Some researchers have studied the antimicrobial activities of NMEO and YKEO. However, due to the different resources and extraction methods, data are still lacking. To our knowledge, the antimicrobial activities of XMEO and PDEO have not been reported. In this study, we tested four citrus EOs on five bacteria, and the results obtained are shown in Table 2 and Table 3. The filter paper diffusion method was used to test the antibacterial activity of EOs against different bacteria, and the activities of EOs were evaluated according to the inhibition zone diameter (IZD) and the minimum inhibitory concentration (MIC).

Following the literature criteria [33], for IZD ≤ 8.0 mm, the bacteria were classified as insensitive to the action of EO; for diameters between 8.0–14.0 mm, as moderately sensitive; for diameters between 14.0–20.0 mm, as sensitive; and for diameters ≥ 20.0 mm, as extremely sensitive.

As shown in Table 2, *B. subtilis*, *S. aureus* and *E. coli* were moderately sensitive to NMEO. XMEO exhibited no inhibitory activity against *S. aureus*, *E. coli*, *P. aeruginosa* and *S. typhimurium*, with IZDs < 8 mm. YKEO had very strong inhibitory effects on *E. coli* and *S. typhimurium* with diameters of 21.58 and 25.39 mm, respectively. *B. subtilis* was sensitive to YKEO with an IZD of 19.49 mm. *B. subtilis* and *E. coli* were moderately sensitive to PDEO, with IZDs of 10.99 and 10.77 mm, respectively. The positive control (100 µg/mL of ampicillin) showed different IZDs against different bacteria; however, *P. aeruginosa* displayed resistance to it. The negative control (sterile water) exhibited no inhibitory activity against all bacteria.

Regarding MIC values, as shown in Table 3, YKEO showed the best antimicrobial activity against *B. subtilis* and *E. coli*—with both MIC values being 1.56 µL/mL. The positive control (ampicillin) showed strong activity against most bacteria except *P. aeruginosa.* The negative control (bacteria only in NB and Tween 80 without EO) exhibited no inhibitory activity against all bacteria. The mechanisms of antimicrobial activity of EOs are not fully understood. It was suggested that EOs can diffuse into cells and damage cell membrane structures [34]. The bioactivities of different EOs can be attributed to certain major compounds or to the synergetic effects between different components [20]. Aggarwal et al. reported that limonene and carvone were active against a wide spectrum of pathogenic fungi and bacteria [35]. We noticed that YKEO had relative higher contents of limonene (91.54%) and carvone (0.34%) than the three other citrus EOs tested. The higher activity of YKEO might be partially attributable to its higher proportions of these two compounds. Anton et al. [36] has reported the activity of ampicillin against *Salmonella typhimurium*. They found that the MIC value was pH dependent (0.63 µg/mL at pH 7 and 2.00 µg/mL at pH 8). Our media pH was 7.4 and the MIC value of 1.56 µg/mL was close to their results. Anomohanran et al. [37] have evaluated the sensitivity of *E. coli*, *P. aeruginosa* and *S*. *typhimurium* to various brands of ampicillin. The MIC values were in the range between 3.90 and 62.50 µg/mL.

### 2.3. Antioxidant Activity

Plant EOs have been reported to scavenge the free radicals that lead to cell death and tissue damage and the development of chronic diseases [16,17,18]. Citrus EOs have antioxidant activity which can delay or prevent cell damage caused by physiological oxidants by inhibiting or eliminating the initiation or propagation of excess reactive species and reduce the risk of potential health effects in humans related to oxidative stress or free radicals [19,20]. In order to explore the potential application of our four citrus EOs in food, cosmetic or pharmaceutical industries, measurement of their antioxidant activities is important. Many assays have been developed to test the antioxidant activities of EOs based on different mechanisms [20]. For convenience to compare our results with other literature, we chose two simple and widely used assays, the 2,2-diphenyl-1-picrylhydrazyl (DPPH) and 2,2′-azino-bis(3-ethylbenzthiazoline-6-sulfonic acid) radical (ABTS) assays, for antioxidant study. Both assays involve an electron transfer and the reduction of a colored oxidant; they are easily monitored via spectrophotometer with an appropriate standard for quantifying the antioxidant activity. Briefly, the DPPH assay is based on the reduction of the purple DPPH radical to 1,1-diphenyl-2-picryl hydrazine, whereas the ABTS assay involves the reduction or radical scavenging of a blue/green ABTS radical to a colorless sulfonic acid [38]. Butylated hydroxytoluene (BHT) was selected as the positive control. The IC_50_ values were calculated using IBM SPSS Statistics 23.0.

As shown in Table 4, BHT displayed potent antioxidant activity. The *IC*_50_ values of all citrus EOs were much higher than those of BHT (0.02 mg/mL and 0.01 mg/mL in DPPH and ABTS assays, respectively) [39]. NMEO presented the best antioxidant activity, with *IC*_50_ values of 15.20 mg/mL for the DPPH assay and 0.80 mg/mL for the ABTS assay. YKEO had the worst antioxidant activity, with *IC*_50_ values of 30.01 mg/mL (DPPH) and 6.62 mg/mL (ABTS), respectively. Yi et al. [19] reported the DPPH and ABTS radical-scavenging activity of cold pressed NMEO with *IC*_50_ values of 22.60 and 1.62 mg/mL, respectively, which are higher values than for our hydrodistilled NMEO (15.20 and 0.80 mg/mL, respectively). Farahmandfar et al. [40] evaluated the DPPH radical scavenging activities of Thomson navel orange peel EOs and found that the *IC*_50_ value of fresh peel EO was 7.86 mg/mL. The much higher *IC*_50_ value (29.70 mg/mL) of PDEO might be attributable to a different maturity stage and variety.

Dawidowicz et al. [41] found that the antioxidant properties of an EO do not always depend on the antioxidant activity of its main component. Synergetic, antagonistic and additive effects are very relevant. Yi et al. [19] studied the antioxidant activities of many individual components of EO in DPPH and ABTS assays. Even though limonene is the predominant component of most citrus EOs, it does not contribute much to antioxidant activity. Thymol and γ-terpinene were the most important components found by DPPH and ABTS radical-scavenging assays. In our case, the order of EOs in both assays was: NMEO > XMEO > PDEO > YKEO. As seen in Table 1, thymol was only found in NMEO, and the proportion of γ-terpinene (8.19%) was the highest in NMEO; this might be the reason NMEO had the best antioxidant activity. XMEO contained 5.80% γ-terpinene and displayed better activity than PDEO and YKEO, which did not contain γ-terpinene. It was very interesting that YKEO presented the best antibacterial activity and the worst antioxidant activity among the four citrus EOs tested. The antibacterial activities of EOs might not be related to their antioxidant activities.

## 3. Materials and Methods

### 3.1. Materials

Nanfeng mandarins, Xunwu mandarins and Yangshuo kumquats were purchased from local fruit market in Ganzhou city in Jiangxi Province in November 2019. A physiological drop of Gannan Newhall navel oranges was collected from the orchard of Gannan Normal University in June 2019.

2,2-Diphenyl-1-picrylhydrazyl (DPPH) was purchased from Tokyo Chemical Industry Co., Ltd. (Tokyo, Japan), 2,2′-Azino-bis(3-ethylbenzthiazoline-6-sulfonic acid) (ABTS) and *n*-alkanes (C_8_–C_20_) were purchased from Sigma-Aldrich (St. Louis, MO, USA). Butylatedhydroxytoluene (BHT) was purchased from Macklin, Shanghai, China. Ampicillin (sodium salt) was purchased from Solarbio, Beijing, China. The following microorganisms were purchased from Beijing, China General Microbiological Culture Collection Center (CGMCC): *Escherichia coli* (ATCC25922), *Staphylococcus aureus* (ATCC25923), *Bacillus subtilis* (ATCC6633), *Salmonella typhimurium* (ATCC14028) and *Pseudomonas aeruginosa* (ATCC9207).

### 3.2. Preparation of Citrus EO Samples

Citrus peel was cut to small pieces (200 g) and put into a round-bottomed distillation flask. Sodium chloride (8 g) and distilled water (800 mL) were added to the flask. After extraction by hydrodistillation in a Clevenger-type device for 6 h, the EO was collected, dried with anhydrous sodium sulfate and stored in a dark glass bottle at 4 °C until analysis. The yields of NMEO, YQEO, XMEO and PDEO were 3.02%, 1.32%, 0.82% and 0.22%, respectively.

### 3.3. GC-MS Analyses

The constituents of citrus EOs were analyzed by GC-MS using an Agilent 7890B gas chromatograph coupled with an Agilent mass spectrometer detector (Agilent Technologies, Santa Clara, CA, USA). GC was conducted on a HP-5 column (30.00 m × 0.25 mm × 0.25 µm). Mass spectra were obtained in electron ionization (EI) mode with an electron energy of 70 eV. The injector and detector were operated at 250 and 300 °C, respectively. The temperature program was held at 50 °C for 2 min, increased at 3 °C/min to 80 °C and then increased at 6 °C/min to 250 °C. The EO sample was prepared as EO: hexane = 1:10 (*v*/*v*). The injection volume was 1 μL. The split ratio was 1:10. The solvent delay time was set to 4 min. Identification of the constituents was carried out by comparing their mass spectra with the National Institute of Standards and Technology (NIST, version 2010, U.S. Department of Commerce, Gaithersburg, MD, USA) data library. The retention indices (RI) of the constituents were determined by co-injection of a C_8_–C_20_ *n*-alkane mixture with the hexane diluted EO sample in the GC-MS equipment and analyzing it under the same conditions described above.

### 3.4. Antimicrobial Activity Assays

#### 3.4.1. Bacterial Growth Conditions

The bacterial strains were maintained in nutrient broth at 37 °C. Subsequently, one colony from each culture was inoculated in liquid medium for 18–24 h with shaking (200 rpm) to obtain freshly cultured bacterial suspensions (>10^8^ CFU mL^−1^) for test.

#### 3.4.2. Determination of the Inhibition Zone Diameter (IZD)

EO was tested on five bacterial strains, using the filter paper diffusion method [42]. A suspension of the tested bacteria (10^6^–10^7^ CFU mL^−1^) was spread on the solid media plates. The paper discs (6 mm diameter) were impregnated with 10 μL EO and placed on the agar surface. The plates inoculated with bacterial strains were incubated for 24 h at 37 °C. The inhibition zone diameter (mm) was measured with a caliper. Ampicillin (100 µg/mL) was selected as positive control, and sterile water without EO was used as negative control for IZD. Each test was performed in triplicate on at least three separate experiments.

#### 3.4.3. Determination of Minimum Inhibitory Concentration (MIC)

The minimum inhibitory concentration (MIC) is defined as the lowest concentration of an EO which inhibits the visible bacterial growth after overnight incubation. It was measured according to the method of Ksouda [43] with minor modifications, using sterile 96-well microplates with a final volume of 200 μL per well. Two-fold serial dilution of an EO (dissolved in Tween 80 (1% *v/v*)) was performed in nutrient broth (NB). Then, 20 μL of each bacterial suspension was inoculated. Each well included 100 μL of the EO diluted in NB, 80 μL NB and 20 μL of cell suspension (10^6^–10^7^ CFU mL^−1^). Ampicillin was selected as the positive control. Bacteria only in NB and Tween 80 (1% *v*/*v*) without EO were used as negative control.

### 3.5. Free Radical-Scavenging Capacity

#### 3.5.1. DPPH Radical-Scavenging Assay

The free radical-scavenging activity of EO was measured using the stable radical 2,2-diphenyl-1-picrylhydrazyl (DPPH) assay according to the method of Xu [44] with minor modifications. EO sample was serially diluted to various concentrations in ethanol, respectively, and then, a 0.5 mL of sample was mixed with 2.5 mL of 60 μM DPPH dissolved in ethanol. The mixture was shaken vigorously for 1 h in the dark, and the absorbance was measured at 517 nm against a solvent blank. The DPPH scavenging activity was expressed according to the following equation:

DPPH scavenging activity (%) = (A_C_ − A_S_)/A_C_ × 100 (1)

where A_C_ is the absorbance of DPPH solution as the negative control, and A_S_ is the absorbance of 0.5 mL EO sample in a 2.5 mL DPPH solution. All samples were analyzed in triplicate, and the results are expressed as mean ± standard deviation. The scavenging activity was expressed as the 50% inhibitory concentration (*IC*_50_), which was the sample concentration at which 50% of DPPH radicals were scavenged after incubation.

#### 3.5.2. ABTS Radical-Scavenging Assay

This method was performed as described by Xu [44], based on the capacity of EO to scavenge the 2,2′-azinobis (3-ethylbenzthiazoline-6-sulfonic acid) radical (ABTS). A reaction of 7 mmol/L ABTS and 2.45 mmol/L potassium persulfate in the dark for 16 h at room temperature generated the ABTS radicals. An accurate volume of the ABTS solution was diluted in ethanol to an absorbance of 0.70 ± 0.05 at 734 nm. Then, 2 mL of the diluted ABTS solution was mixed with 100 μL of EO and stood at room temperature for 6 min. The absorbance was then measured at 734 nm. ABTS scavenging activity was calculated using the following equation:

ABTS scavenging activity (%) = (A_C_ − A_S_)/A_C_ × 100 (2)

where A_C_ is the absorbance of the ABTS solution as the control and A_S_ is the absorbance of the ABTS solution after adding the EO sample. The *IC*_50_ was calculated from the graph of scavenging percentage against EO concentration using IBM SPSS Statistics 23.0. The results are expressed as mean ± standard deviation of three experiments.

### 3.6. Statistical Analysis

All GC-MS experiments, and antimicrobial and antioxidant tests were carried out in triplicate. The results in Table 1, Table 2 and Table 4 were expressed as mean ± standard deviation (S.D.). Data obtained were analyzed by one way analysis of variance (ANOVA) followed by Tukey’s post hoc tests using IBM SPSS Statistics 23.0. (IBM Corp. Released 2015. IBM SPSS Statistics for Windows, Version 23.0. Armonk, NY, USA). In Table 1, the proportions of each compound in the different EO samples were compared. In Table 2, the inhibition zone diameters of four citrus EOs and ampicillin against each bacterium were compared. In Table 4, *IC*_50_ of four citrus EOs and BHT using the same assay were compared.

## 4. Conclusions

China is one of the world’s largest producers of citrus fruit, and the beneficial roles of citrus EOs have been widely reported. However, the chemical composition and bioactivities of some citrus EOs produced in southern China have not been well studied. In this study, four types of citrus EOs were extracted via hydrodistillation method, and their chemical compositions, and antimicrobial and antioxidant activities were studied. YKEO showed strong antibacterial activity against *B. subtilis*, *E. coli* and *S. typhimurium*, with MIC values ranging from 1.56 to 6.25 µL/mL. All citrus EOs scavenged DPPH and ABTS free radicals in a dose-dependent manner. The two mandarin EOs, NMEO and XMEO, displayed better antioxidant activity than the other two citrus EOs. The results showed that some citrus EOs have potential for future utilization in the pharmaceutical and food industries.

## Figures and Tables

**Table 1:** Chemical compositions of the citrus EOs as found by GC-MS.

No.	RI _exp._	RI _lit._	Compounds	Composition (%)				CharacteristicMass Ions (*m*/*z*)
				NMEO	XMEO	YKEO	PDEO	
1	923	927	α-Thujene	0.29 ± 0.00 ^a^	0.15 ± 0.01 ^b^	–	–	77, 91, 92, 93
2	929	929	α-Pinene	1.22 ± 0.03 ^a^	0.83 ± 0.05 ^b^	0.62 ± 0.07 ^c^	0.33 ± 0.01 ^d^	39, 77, 91, 93
3	970	963	Sabinene	0.35 ± 0.00 ^a^	0.08 ± 0.01 ^c^	0.15 ± 0.02 ^b^	0.33 ± 0.01 ^a^	77, 91, 93, 136
4	972	970	β-Pinene	2.00 ± 0.05 ^a^	0.46 ± 0.02 ^b^	–	0.09 ± 0.01 ^c^	41, 69, 91, 93
5	990	992	β-Myrcene	2.08 ± 0.01 ^b^	2.02 ± 0.12 ^b^	2.73 ± 0.22 ^a^	1.90 ± 0.04 ^b^	39, 41, 69, 93
6	1003	1002	Octanal	0.29 ± 0.03 ^a^	–	–	–	41, 43, 44, 56
7	1004	1006	α-Phellandrene	–	0.10 ± 0.00 ^b^	–	0.17 ± 0.03 ^a^	77, 91, 92, 93
8	1009	1007	3-Carene	–	–	–	0.08 ± 0.00 ^a^	77, 79, 91, 93
9	1016	1020	α-Terpinene	0.21 ± 0.01 ^b^	0.14 ± 0.01 ^c^	–	0.26 ± 0.01 ^a^	91, 93, 121, 136
10	1030	1029	Limonene	79.13 ± 0.41 ^d^	86.03 ± 0.29 ^c^	91.54 ± 1.22 ^a^	88.25 ± 0.62 ^b^	67, 68, 79, 93
11	1060	1061	γ-Terpinene	8.19 ± 0.28 ^a^	5.80 ± 0.07 ^b^	–	–	77, 91, 93, 136
12	1088	1089	Terpinolene	0.49 ± 0.04 ^a^	0.33 ± 0.02 ^b^	–	0.18 ± 0.01 ^c^	93, 105, 121, 136
13	1101	1102	Linalool	1.81 ± 0.04 ^a^	0.43 ± 0.03 ^c^	0.12 ± 0.02 ^d^	1.50 ± 0.06 ^b^	41, 55, 69, 93
14	1122	1123	*trans-p*-Mentha-2,8-dien-1-ol	–	–	0.18 ± 0.00 ^a^	0.06 ± 0.02 ^b^	43, 79, 109, 134
15	1134	1136	*cis*-Limonene oxide	0.03 ± 0.00 ^b^	0.02 ± 0.00 ^b^	0.31 ± 0.01 ^a^	0.03 ± 0.00 ^b^	41, 43, 67, 93
16	1139	1139	*trans*-Limonene oxide	0.05 ± 0.00 ^b^	0.03 ± 0.01 ^c^	0.20 ± 0.01 ^a^	0.02 ± 0.01 ^c^	43, 67, 94, 108
17	1141	1145	*cis*-β-Terpineol	–	–	–	0.03 ± 0.01 ^a^	41, 43, 71, 93
18	1147	1151	*trans*-β-Terpineol	–	–	–	0.14 ± 0.02 ^a^	43, 71, 93, 136
19	1155	1155	Citronellal	–	0.01 ± 0.01 ^b^	–	0.03 ± 0.00 ^a^	41, 55, 69, 95
20	1177	1178	Terpinen-4-ol	0.22 ± 0.01 ^b^	0.14 ± 0.02 ^bc^	0.06 ± 0.00 ^c^	1.98 ± 0.07 ^a^	71, 77, 91, 93
21	1190	1191	α-Terpineol	0.45 ± 0.02 ^b^	0.25 ± 0.02 ^c^	0.07 ± 0.01 ^d^	1.01 ± 0.04 ^a^	59, 68, 79, 93
22	1203	1204	Decanal	0.57 ± 0.09 ^a^	–	–	0.25 ± 0.02 ^b^	41, 43, 55, 57
23	1220	1224	*trans*-Carveol	–	–	0.30 ± 0.04 ^a^	0.17 ± 0.03 ^b^	41, 55, 84, 109
24	1231	1232	Citronellol	–	–	–	0.17 ± 0.04 ^a^	41, 67, 69, 81
25	1233	1238	*cis*-Carveol	–	–	0.22 ± 0.04 ^a^	–	91, 105, 119, 134
26	1243	1242	Neral	–	–	–	0.12 ± 0.03 ^a^	27, 39, 41, 69
27	1246	1251	Carvone	0.05 ± 0.03 ^b^	–	0.34 ± 0.05 ^a^	–	39, 54, 82, 93
28	1253	1253	Geraniol	–	–	–	0.04 ± 0.01 ^a^	41, 68, 69, 93
29	1273	1272	Geranial	–	–	–	0.23 ± 0.13 ^a^	39, 41, 69, 84
30	1277	1276	Perillyl aldehyde	0.09 ± 0.01 ^a^	0.04 ± 0.01 ^b^	0.09 ± 0.01 ^a^	–	39, 67, 68, 79
31	1301	1295	Thymol	0.18 ± 0.04 ^a^	–	–	–	91, 115, 135, 150
32	1309	1310	Undecanal	–	0.03 ± 0.02 ^a^	–	–	41, 43, 55, 57
33	1340	1339	δ-Elemene	0.04 ± 0.00 ^b^	0.10 ± 0.00 ^a^	–	–	41, 93, 121, 136
34	1366	1371	Neryl acetate	–	0.03 ± 0.01 ^b^	0.07 ± 0.03 ^a^	0.02 ± 0.01 ^b^	41, 68, 69, 93
35	1379	1375	Copaene	–	0.07 ± 0.01 ^a^	0.06 ± 0.02 ^a^	0.02 ± 0.01 ^b^	93, 105, 119, 161
36	1386	1382	Geranyl acetate	–	–	0.25 ± 0.09 ^a^	–	41, 69, 79, 93
37	1395	1395	β-Elemene	0.04 ± 0.00 ^bc^	0.52 ± 0.02 ^a^	0.08 ± 0.02 ^b^	0.01 ± 0.01 ^c^	41, 68, 81, 93
38	1410	1411	Dodecanal	0.09 ± 0.02 ^a^	–	–	0.02 ± 0.01 ^b^	41, 43, 55, 57
39	1424	1428	β-Caryophyllene	0.02 ± 0.00 ^c^	0.09 ± 0.01 ^a^	0.05 ± 0.01 ^b^	0.03 ± 0.00 ^c^	41, 69, 79, 93
40	1437	1431	γ-Elemene	–	–	0.02 ± 0.01 ^a^	–	79, 93, 107, 121
41	1439	1438	Perillyl acetate	–	–	0.03 ± 0.01 ^a^	–	43, 68, 91, 119
42	1459	1455	α-Humulene	–	0.10 ± 0.02 ^a^	0.02 ± 0.01 ^bc^	0.03 ± 0.00 ^b^	41, 80, 93, 121
43	1480	1478	β-Selinene	–	0.03 ± 0.01 ^a^	–	–	41, 93, 105, 107
44	1486	1485	Germacrene D	0.09 ± 0.01 ^b^	0.22 ± 0.05 ^a^	0.27 ± 0.06 ^a^	0.02 ± 0.00 ^b^	105, 119, 161, 204
45	1492	1499	α-Selinene	–	0.03 ± 0.01 ^a^	–	–	161, 175, 189, 204
46	1502	1500	Valencene	0.09 ± 0.01 ^ab^	0.28 ± 0.07 ^a^	–	0.19 ± 0.01 ^bc^	147, 161, 189, 204
47	1511	1504	α-Farnesene	1.50 ± 0.17 ^a^	0.69 ± 0.18 ^b^	0.04 ± 0.01 ^c^	0.04 ± 0.01 ^c^	41, 55, 69, 93
48	1530	1527	δ-Cadinene	0.03 ± 0.01 ^b^	0.16 ± 0.04 ^a^	0.04 ± 0.02 ^b^	0.07 ± 0.00 ^b^	134, 161, 189, 204
49	1564	1560	Germacrene B	–	0.04 ± 0.01 ^ab^	0.08 ± 0.02 ^a^	–	93, 105, 107, 121
50	1568	1565	*trans*-Nerolidol	–	–	0.04 ± 0.01 ^a^	–	41, 43, 69, 93
51	1586	1583	Caryophyllene oxide	0.03 ± 0.00 ^a^	–	–	–	41, 79, 91, 105
52	1640	1643	α-Eudesmol	–	–	–	0.03 ± 0.01 ^a^	59, 149, 161, 189
53	1651	1651	Cubenol	–	0.04 ± 0.01 ^a^	–	–	119, 161, 189, 204
54	1664	1661	β-Eudesmol	–	0.12 ± 0.03 ^a^	0.12 ± 0.02 ^a^	0.04 ± 0.01 ^b^	59, 108, 149, 164
55	1762	1756	α-Sinensal	–	–	–	0.16 ± 0.04 ^a^	55, 79, 93, 134
Total				99.63	99.41	98.10	98.05	
Monoterpene hydrocarbons				93.96	95.94	95.04	91.59	
Oxygenated monoterpenes				2.88	0.95	2.24	5.55	
Sesquiterpene hydrocarbons				1.81	2.33	0.66	0.41	
Oxygenated sesquiterpenes				0.03	0.16	0.16	0.23	
Others(straight-chain aldehydes)				0.95	0.03	0.00	0.27	

RI _exp._, experimental retention indices determined on a HP-5 column, using the homologous series of *n*-alkanes (C_8_–C_20_). RI _lit._, literature retention indices on similar columns. “–“, not detected. Results are expressed as mean area percentage (%) ± standard deviation (S.D.) of three independent determinations in triplicate (*n* = 3). Means in the same row followed by different superscript letters are significantly different (*p* < 0.05) according to one-way analysis of variance (ANOVA) followed by Turkey’s post hoc test (the proportion of a compound which was not detected was set to be 0.00).

**Table 2:** Inhibition zone diameters (IZD, mm) of citrus EOs and ampicillin.

Bacterial Strain	Essential Oil				Ampicillin (100 µg/mL)
	NMEO	XMEO	YKEO	PDEO	
*Bacillus subtilis* (G+)	10.57 ± 0.74 ^cd^	8.49 ± 0.41 ^d^	19.49 ± 1.41 ^b^	10.99 ± 1.02 ^c^	24.05 ± 0.78 ^a^
*Staphylococcus aureus* (G+)	9.54 ± 1.19 ^b^	6.90 ± 0.63 ^c^	8.02 ± 0.16 ^bc^	7.66 ± 0.41 ^c^	33.03 ± 0.24 ^a^
*Escherichia coli* (G−)	9.48 ± 1.22 ^bc^	7.44 ± 0.13 ^c^	21.58 ± 1.57 ^a^	10.77 ± 1.33 ^b^	7.95 ± 0.21 ^bc^
*Pseudomonas aeruginosa* (G−)	7.93 ± 0.49 ^a^	6.15 ± 0.05 ^b^	8.42 ± 0.66 ^a^	6.30 ± 0.13 ^b^	6.00 ± 0.00 ^b^
*Salmonella typhimurium* (G−)	6.35 ± 0.23 ^c^	6.00 ± 0.00 ^c^	25.39 ± 1.25 ^a^	6.00 ± 0.00 ^c^	13.20 ± 0.30 ^b^

Zone of growth inhibition values are presented as mean ± standard deviation for at least three experiments in mm, including the 6.0 mm disk diameter. Means in the same row followed by different superscript letters are significantly different (*p* < 0.05) according to one-way analysis of variance (ANOVA) followed by Turkey’s post hoc test.

**Table 3:** Minimum inhibitory concentrations (MIC).

Bacterial Strain	Essential Oil (µL/mL)				Ampicillin (µg/mL)
	NMEO	XMEO	YKEO	PDEO	
*Bacillus subtilis* (G+)	6.25	12.50	1.56	6.25	25.00
*Staphylococcus aureus* (G+)	25.00	50.00	12.50	50.00	0.10
*Escherichia coli* (G−)	25.00	12.50	1.56	6.25	3.12
*Pseudomonas aeruginosa* (G−)	25.00	50.00	100.00	100.00	800.00
*Salmonella typhimurium* (G−)	25.00	100.00	6.25	50.00	1.56

Experiments were carried out in triplicate.

**Table 4:** Antioxidant activities of citrus EOs by DPPH and ABTS assays.

Samples	DPPH *IC*_50_ (mg/mL)	ABTS *IC*_50_ (mg/mL)
NMEO	15.20 ± 1.85 ^b^	0.80 ± 0.05 ^d^
XMEO	18.25 ± 0.74 ^b^	1.64 ± 0.18 ^c^
YKEO	30.01 ± 1.14 ^a^	6.62 ± 0.24 ^a^
PDEO	29.70 ± 1.02 ^a^	4.17 ± 0.09 ^b^
BHT	0.02 ± 0.01 ^c^	0.01 ± 0.00 ^e^

*IC*_50_ are the averages of triplicate experiments and are represented as mean ± standard deviation. Means in the same column followed by different superscript letters are significantly different (*p* < 0.05) according to one-way analysis of variance (ANOVA) followed by Turkey’s post hoc test.

## Data Availability

Not applicable.

## Sample Availability

Samples of the four citrus EOs are available from the authors.
